# Using Digital Technology for Sexual and Reproductive Health: Are Programs Adequately Considering Risk?

**DOI:** 10.9745/GHSP-D-19-00239

**Published:** 2019-12-23

**Authors:** Loraine J. Bacchus, Kate Reiss, Kathryn Church, Manuela Colombini, Erin Pearson, Ruchira Naved, Chris Smith, Kathryn Andersen, Caroline Free

**Affiliations:** a London School of Hygiene & Tropical Medicine, London, England.; b Marie Stopes International, London, England.; c Ipas, Chapel Hill, NC, USA.; d icddr,b, Dhaka, Bangladesh.; e School of Tropical Medicine and Global Health, Nagasaki University, Nagasaki, Japan.

## Abstract

Digital technologies provide opportunities for advancing sexual and reproductive health and services but also present potential risks. We propose 4 steps to reducing potential harms: (1) consider potential harms during intervention design, (2) mitigate or minimize potential harms during the design phase, (3) measure adverse outcomes during implementation, and (4) plan how to support those reporting adverse outcomes.

## INTRODUCTION

Health care is increasingly being delivered through digital channels such as the internet, mobile phone messaging, social media, apps, voice, video messaging, and telemedicine. This trend has been facilitated by diffusion of mobile technology and rapid advances in artificial intelligence. Digital communication channels offer wide coverage, allow messaging to be targeted to particular groups or individuals, and offer potential for enhancing the delivery of sexual and reproductive health and rights (SRHR) information and support.

Recent developments in the SRHR field include the provision of online testing for sexually transmitted infections (STIs) that have been shown to almost double the uptake of STI tests^1^ and e-contraception whereby the oral contraceptive pill can be ordered online.^2^ Telemedicine in sexual and reproductive health (SRH) can overcome geographic or social and behavioral barriers to accessing services and facilitate self-use of products or services.^3^ It has been used to support medication abortion and facilitate distribution of abortifacient pills backed up by remote care and support.^4^ Interventions targeting a range of populations and SRHR topics across different cultural contexts have been shown to be acceptable to the end user and feasible to implement.^4^^–^^8^ Interventions can be designed to be accessible across socioeconomic groups and to those at high risk.^2^^,^^9^^,^^10^ Improvements in knowledge and contraceptive or health-seeking behavior have been demonstrated.^6^^,^^11^^–^^13^ However, not all studies show benefits, as exemplified by the Reiss et al. intervention in Bangladesh that showed no effect on contraceptive use.^14^

### Sensitivity of SRH and SRH Services

SRH and SRH services are highly sensitive. There can be considerable social disapproval of sexual behaviors within some groups, such as adolescents and men who have sex with men, or outside of marriage. HIV and other STIs are stigmatized and decisions about fertility are highly influenced by partners as well as by members of the broader family and community. Failure to keep SRH service use confidential or disclosure of HIV/STI status can result in conflict with or loss of support of parents, stigma, blame, discrimination, or new or escalating verbal or physical violence.^15^^–^^17^ Women in many settings, in particular those living in more patriarchal, socially conservative contexts, are also often victims of reproductive coercion, through which their autonomy over reproductive choice is greatly impaired.^18^^–^^19^ Women’s requests to use contraception can result in suspicions of infidelity, and many women fear violence if they request to control their own fertility.^20^ In this context, a mechanism by which harm may occur to women is through breaches of confidentiality. If a partner or family member becomes aware of the woman’s access to SRH services, they may restrict the woman from returning to a health facility and accessing health care.

Reproductive coercion is a form of intimate partner violence (IPV), which includes physical, sexual, and emotional abuse and controlling behaviors by a current or former partner.^21^ IPV is one of the most common forms of violence against women with 1 in 3 women globally having experienced it in their lifetime.^22^ The estimated prevalence of reproductive coercion among women experiencing IPV is 8% to 16% in U.S. studies.^23^^–^^24^ Negative SRH outcomes, such as unintended pregnancy and termination of pregnancy, are potential consequences of reproductive coercion.^25^^–^^28^ A study in Bangladesh found that IPV was associated with women coming to the health facility alone for menstrual regulation rather than being accompanied by their husband or partner.^29^

These issues have rendered privacy and confidentiality a key tenet of quality SRH information and services. They are especially important for protecting vulnerable women and those experiencing IPV.

### Potential Risks of Digital SRH Information Support and Services

Although the extensive use of digital technologies affords opportunities for SRH promotion and service provision, interventions that reach into users’ homes and personal spaces may also entail particular risk. For example, interventions designed to increase adherence to antiretroviral medication for patients who have HIV may inadvertently result in disclosure of HIV status. Interventions promoting contraception may inadvertently disclose contraceptive use that others may not condone.^14^

Mobile phones have features that can be used to enhance privacy. In SRH contexts, sensitive personal content delivered by digital media can be confidential, especially if phone features such as passwords are not shared and alerts are switched off.^9^ However, boundaries in relation to mobile phone access and concepts of privacy vary between individuals and contexts. Phones may be shared within families, hindering the potential to deliver sensitive personal information.^30^ Women in abusive situations may have their digital media use controlled by their partner and family members, or digital media may be used to perpetrate abuse (i.e., harass, stalk, and monitor).^31^

## MINIMIZING AND RESPONDING TO RISKS OF HARM IN SRH DIGITAL INTERVENTIONS

As with other forms of communication and service delivery in SRHR, potential harms of using digital technology must be first considered, then mitigated or minimized in the intervention design, and measured during evaluation or implementation. Those who deliver interventions should also plan how to respond to reports of harm. We discuss 4 key steps that should be taken to minimize and respond to risk of harm in SRHR interventions, specifically focusing on digital interventions (Box).

BOX Steps to Reduce Potential Harms in Providing Sexual and Reproductive Health Services

1. Consider potential harms during intervention design.With input from users and key stakeholders:
  - Develop theoretical frameworks to elaborate mechanisms through which adverse outcomes may occur.
  - Understand how digital media are used, shared, and kept private.
  - Understand the sensitivity, stigma, and social and power dynamics influencing SRH in the context for which the intervention is planned.
2. Mitigate or minimize potential harms in the design phase.
  - Consult with potential users in intervention development to determine whether privacy can be achieved. Consider the mode of delivery and intervention features required to afford privacy and confidentiality, when wanted.
  - Test and refine interventions with input from users receiving the intervention as planned.
3. Measure adverse outcomes.
  - Use research methods to reduce reporting bias such as by using standardized, validated measures.
  - Follow ethical guidelines for conducting research on violence.
4. Plan how to support those reporting adverse events, including IPV.
  - Provide links to existing services and/or staff training according to setting.

### 1. Consider Potential Harms

It is common practice to develop theories to explain how intended effects of interventions may occur and to explore these in evaluations. This practice can inform understanding as to why interventions did or did not work, what the active components were, and how they may be transferred to other contexts or populations. In addition to identifying the mechanisms behind intended benefits, it is also important to identify “dark logic” models^32^ (i.e., the causal pathways from interventions to potential adverse outcomes). Mechanisms for surfacing potential harms include consulting key stakeholders at an early stage of intervention development who have deep insight into how interventions work in local contexts. Other mechanisms include building a comparative understanding of potential harm across similar interventions through a review of the evidence (where such an evidence base exists) and reflecting on how broader sociocultural, political, and economic forces may constrain recipients of the intervention (providers, clients) and cause unintended negative consequences.^32^ For digital intervention design, key stakeholder interviews must include interviews with potential users to understand how digital media are used and shared and whether they could be kept private. It is also important to understand the sensitivity, stigma, social, and power dynamics influencing SRH in the intervention context. Given the infrequency of many negative outcomes, it may also be important to consider the use of meta-analysis to understand the existing evidence about potential risk, since individual studies may remain underpowered to detect effects.^32^^–^^33^

### 2. Mitigate and Minimize Harms

In conjunction with users, those designing interventions should consider which risk reduction strategies are needed. For interventions delivered by digital media, the first consideration is the choice of media for communication. Designers should consider the balance between the convenience of the digital intervention, maintenance of privacy and confidentiality, cultural acceptability, and effectiveness of the intervention. These factors will differ between contexts. For example, the Reiss et al. intervention in Bangladesh used voice messages^14^ because participants in their formative research indicated that they felt voice messages were more private as they do not remain on the phone.^34^ However, for some, voice messages may be more intrusive than text messages or other digital media because recipients have to answer the call to receive the message irrespective of where they are, what they are doing, or who they are with. In some contexts, use of text messages is more common than voice calls, thereby arousing less suspicion.

Once designers select the digital media, they can further minimize risk by using a range of options.^35^ Where privacy cannot be achieved, designers may opt to send content specifically designed for sharing. For example, in work that we conducted in Cambodia^36^ targeting factory workers, the sensitivity of sexual activity among unmarried female factory workers and shared living arrangements led to the intervention promoting contraceptive use and services via social media videos designed to be viewed alone or shared with groups of friends. Alternatively, designers may opt to send general content rather than personalized information that may reveal confidential behaviors. The use of general content has been shown to be both effective in changing attitudes and safe in 3 different settings.^37^^–^^38^

Further strategies to prevent harm in the case of content being viewed by others include careful naming of apps or wording of notifications, using discreet icons, avoiding stating the source within each message, and using general or untraceable telephone numbers rather than a short code.^9^ Strategies to facilitate privacy include using passwords, blocking alerts, and using protective firewalls for sensitive applications. Other features that can protect recipients from having content viewed by others include escape buttons on websites and apps that allow pages to be shut quickly or switched to other sites^39^ and customizable privacy settings including for data storage.^9^ For example, interventions may be designed with data-purging mechanisms and without unnecessary automatic data collection systems that are often included as standard features.^35^ Recipients can also be advised to delete messages or search histories if they have concerns about privacy.^9^ Timing of content delivery can be critical to ensure messages are only sent when privacy can be assured. In a messaging intervention for sex workers, potential recipients only wanted to receive messages on Saturday mornings when they were not working.^40^ In Bolivia, Palestine, Tajikistan, and the UK, where people have opted to receive push content (written messages sent to their phones), recipients reported them to be highly acceptable, safe, and effective in changing attitudes, intentions, and some preventative behaviors.^9^^,^^38^^,^^41^^,^^42^ In other circumstances or contexts it may only be considered appropriate to use pull content (only sending messages or content on request) to reduce the potential for harm or it may be necessary to avoid outbound messages altogether.^43^

After considering the above factors early in the design phase, designers should test intervention prototypes and content with users. This phase should involve delivering the intervention in real time as intended and seeking feedback about recipients’ experiences of the intervention, its acceptability, intrusiveness, ability to keep content private when wanted, and perceived impacts on the knowledge, attitudes, and behaviors targeted. In our previous interventions, many recipients wanted and chose to share content with siblings, partners, or mothers, but they felt it was important to have the ability to keep content private when they chose.^8^^,^^38^^,^^41^^,^^42^

### 3. Measure Potential Harms

Measuring potential risks is key to establishing the success of steps taken to mitigate or minimize harms. Research offers critical opportunities to assess potential harms. Yet adverse event measurement is often limited to clinical research studies and remains rare and inconsistent in social and behavioral intervention evaluation.^32^^,^^33^^,^^44^^,^^45^ Despite the known association between SRH or SRH service use and stigma, blame, discrimination, new or escalating verbal or physical violence from partners, family, or others (including violence during pregnancy, abortion, or post-HIV diagnosis),^46^^–^^49^ it is surprising how few SRHR intervention trials have evaluated the impact of interventions on potential harms. For example, of the 5 trials included in a systemic review of mobile phone-based interventions for improving contraceptive use, only 1 assessed unintended adverse outcomes.^11^ In a systematic review of STI/HIV partner notification, 5 of 26 studies assessed the number of harmful events reported.^50^ Nonetheless, where evaluated, rare but important harms have been recorded such as perceived breaches of confidentiality regarding previously undisclosed HIV status.^51^

The importance of measurement is illustrated by the Reiss et al. intervention.^14^ In the intervention development, the researchers considered the potential for harm among a group of women seeking abortion in a context where partner violence is common, and they took a number of steps to mitigate harm.^14^ The research involved a substantive formative phase to develop and define the intervention. This informed the authors’ understanding of user needs during the post-menstrual regulation period and the acceptability of having information delivered on this topic via mobile phone.^14^^,^^34^ During in-depth interviews, user inputs were sought on the message content, mode of delivery, and issues related to confidentiality and privacy where prototype messages were played and shown to participants. Concerns were not raised at that stage by participants.^34^ User-testing involved sharing draft content with contraceptive users.^14^ During the trial recruitment process, researchers played an example of message content to all participants to check that they were comfortable receiving intervention voice messages.^14^ The subsequent evidencing of the IPV findings in the Reiss randomized controlled trial demonstrates that even in-depth formative work and screening may be insufficient to remove risk.^14^ This may reflect the challenges in overcoming social desirability reporting bias or power imbalances, or the challenges of asking users about hypothetical untested scenarios during the development phase. A pilot test of the intervention delivered as planned, followed by in-depth interviews, may have been more likely to reveal harms associated with the intervention, although the sample sizes of a pilot still would not have determined the degree of risk. However, measurement in the randomized controlled trial was key in preventing the scale-up of an intervention with no benefits, but with risk of harm. It is essential that the unintended consequences of all SRHR interventions, digital or otherwise, be consistently collected and reported on.

#### Challenges to Measuring Intimate Partner Violence

Given the documented links described earlier between abuse (including IPV) and SRH outcomes, reproductive coercion and other forms of abuse should be measured consistently in SRHR interventions. The use of valid and reliable measures will permit comparative analysis between settings and types of intervention. Although valid and reliable measures of IPV exist, the task of measuring IPV is complicated. Studies of IPV commonly use multicomponent self-report measures describing specific behaviors, but many validated measures are too long to be included in surveys, particularly if IPV is not the focus. Psychological and emotional abuse are particularly challenging to measure.^52^^–^^54^ Due to the insidious nature of coercive and controlling behavior, women may not label them as abusive or misinterpret survey items leading to underreporting.^55^ They may also be reluctant to identify with some forms of abuse. For example, in the context of a relationship where there has been prior consensual sex, a woman may be more hesitant to label an event as coerced or forced sex, even if consent was not given.^56^ However, even physical violence, the most tangible form of violence, is difficult to measure in different contexts and tools vary in their comprehensiveness, affecting calculated prevalence rates.^53^

Advances in overcoming reporting bias, such as the collection of contextual data and outcomes (e.g., injury or other impacts),^54^^,^^57^^,^^58^ may be ethically and practically challenging for wider SRHR evaluation where IPV measurement is not the primary objective. Lengthy surveys may compromise broader data quality by reducing response rates.^59^ It is advisable to augment quantitative evaluation of IPV with qualitative methods to enhance interpretation and improve the reliability and validity of the findings, as well as explore unanticipated harmful outcomes post-intervention.^60^^–^^61^ Clearly, there is a need to find balance between including questions on key dimensions of IPV while being mindful of these ethical or practical concerns. The Bangladesh trial used closed-answer questions about specific acts of violence and followed the quantitative phase with in-depth interviews exploring the violence outcome.^14^ The World Health Organization developed ethical and safety guidelines for researching violence against women, given the inherent risks to participants and researchers.^62^^,^^63^ Recommendations include researcher training (i.e., for interviewing, using safety protocols, and dealing with participant distress and requests for help), provision to participants of up-to-date referral information for good-quality local organizations offering support to women experiencing violence,^63^ and provision of psychosocial support for researchers experiencing vicarious trauma.^64^ However, a pragmatic approach may be necessary as implementation of the guidelines requires additional resources in terms of time, finances, staffing, and technical support. This may include using a combination of remote and in-country research capacity strengthening sessions with local researchers. An advisory group with expertise in gender-based violence and intervention research can review safety and distress protocols for researchers and participants, as well as offer guidance when potential issues of harm and adverse events arise as a result of the research. Community-based organizations offering support should be identified, alerted to the intervention, and prepared for the potential increase in referrals as a result of the study.

### 4. Plan How to Support Women Experiencing IPV

Although digital interventions pose potential risks, they also provide significant opportunities for women experiencing IPV. For example, in clinical settings that lack privacy and where face-to-face communication can be overheard, the use of technology can reduce the risk of harm resulting from breaches of confidentiality. For some individuals, such interventions may be the only feasible way to obtain information and services. SRH services offer an entry point where women can safely disclose IPV experiences to their health care provider and access support and referrals within and outside of the health system.^65^^–^^67^ Technology may also offer a greater sense of anonymity, which can reduce the anticipated stigma associated with direct disclosure to a health care provider.^68^ The potential also exists to identify women experiencing IPV through digital SRHR outreach (information, mobile services) and streamline their referral for counseling and support. Evidence-based global guidelines on management of IPV state that health care providers should receive training in how to identify clients affected by IPV and provide first-line support that includes empathic listening, psychosocial support, and referral to appropriate services.^69^ However, at present, evidence of effective interventions for addressing IPV in SRH is limited, particularly for low- and middle-income countries, and focus mainly on antenatal care settings.^69^ Similarly, there has been little investigation into whether digital SRHR interventions can be effective in providing support for individuals who are experiencing IPV. This support has been requested during development of a text message intervention designed to reduce unintended pregnancy among female sex workers in Kenya, where many participants expressed the need for a service to report violence and receive emergency assistance. The intervention was adapted to incorporate an automatic response to any direct message into the system and information on gender-based violence support services.^40^

In recent years, there has been a proliferation of web-based apps that provide information about IPV, for example, on services and healthy relationships, as well as interactive tools for assessing risk and developing safety plans.^70^^–^^72^ These apps have primarily targeted women in the general population, with the exception of 1 study, a nurse home visitation program for pregnant and postnatal women using mHealth.^68^

Additionally, the evidence is skewed toward high-income countries (i.e., Australia, Canada, New Zealand, and the United States), and little is known about how well these technologies work in low- and middle-income settings where there are greater structural barriers to women seeking help. Also, it is not clear how these apps may be adapted for use in SRH settings. Jewkes et al. (2019) highlights the need for more formative research and evaluation of their efficacy before roll out.^73^ This research should include qualitative exploration of women’s views, particularly their comfort in using apps to access information and support and their preference in engaging with apps alone versus with a health care provider. Research on health care providers’ views should also include factors they should consider when trying to enroll women who are experiencing IPV into studies using apps. Cognitive interviewing techniques can be used to improve content, understandability, and design features. Finally, pilot and proof-of-concept studies are needed before progressing to trials or observational studies.

Whether addressing violence is the primary objective of an intervention or a component in a broader SRH intervention, the potential for this harm needs to be considered. In violent relationships, where abusive partners may search mobile phones and browsing histories, accessing support for violence or SRHR interventions could trigger a violent episode.^73^ The need to protect women and children, ensure their safety, and avoid the risk of retaliation while they seek help is critical. Further research is needed to understand the relative acceptability, risks, and effectiveness of different models.

## CONCLUSION

As mobile phone networks proliferate throughout low- and middle-income countries, digital technologies offer huge potential to support women to achieve positive SRHR outcomes. Technology can make information and services available when and where they are needed, and can facilitate a broader shift toward user-controlled products and services, including for family planning. However, delivering SRHR services and offering information and support to empower women to take control of their health and fertility may, in some instances, pit individuals’ sexual health rights and reproductive autonomy against conservative or patriarchal social norms; this conflict may in turn place some women at risk. Nevertheless, digital technologies have potential to do good if they are well designed and implemented because they are highly accessible and can allow scale to be achieved at low cost.

We recommend that program designers follow 4 steps to reduce potential harms (Box 1). Taking these steps to develop interventions that are safe by design is key to the proliferation of digital SRH information, support, and services.

## Notes

**Peer Reviewed**
